# Psychopathology and literary creativity: a pathographical mixed-methods analysis of the tang dynasty poet Li Bai

**DOI:** 10.3389/fnhum.2026.1893177

**Published:** 2026-09-28

**Authors:** Chunfeng Xiao, Yufei Wang, Junjie Chen, Wenqi Geng, Jinya Cao, Jing Wei

**Affiliations:** 1 Department of Psychological Medicine, Peking Union Medical College Hospital, Chinese Academy of Medical Science & Peking Union Medical College, Beijing, China; 2 Chinese Academy of Medical Science & Peking Union Medical College, Beijing, China

**Keywords:** bipolar disorder, creativity, Li Bai, poetry, psychopathology

## Abstract

**Background:**

The Tang dynasty poet Li Bai (701–762 CE) is renowned for extraordinary literary creativity. Traditional historical perspectives frequently link his “Poet Immortal” reputation to alcohol-associated euphoria. We examined whether temporal fluctuations in his creative output co-occurred with biographical and textual features compatible with mood-spectrum psychopathology, while considering alcohol use and major life events as alternative explanations.

**Methods:**

This retrospective pathographical mixed-methods study analyzed Li Bai’s literary corpus and biographical archives. Annual creative output was quantified for 836 securely dated poems across a 48-year career. Years exceeding 2 SDs above the career mean were used as data-derived anchors for episode-centered qualitative review. We additionally calculated the proportion of the corpus produced in peak years and a peak-to-nonpeak productivity ratio. Literary and historical evidence was then mapped to DSM-5 mood-episode symptom domains, with explicit attention to temporal fit and competing situational explanations.

**Results:**

Annual productivity showed three extreme-output years: 743 CE (*n* = 73, z = 3.29), 753 CE (*n* = 56, z = 2.29), and 759 CE (*n* = 63, z = 2.70). These 3 years (6.25% of career years) contained 192 poems (23.0% of the securely dated corpus); mean output was 64.0 poems/year in peak years versus 14.3 poems/year in the remaining years (ratio, 4.47). Episode-centered review found the strongest convergence of elevated or expansive affect, grandiose self-appraisal, and reckless behavior around 742–743 and 752–753 CE. The 758–759 CE episode included marked elation after an imperial pardon, although this event provides a strong nonpathological explanation for the affective shift. Depressive and mixed-affect expressions were also present. The temporal convergence is compatible with, but does not establish, a bipolar-spectrum interpretation or a causal effect of mood symptoms on creativity.

**Conclusion:**

Li Bai’s creative output was highly episodic, and the identified productivity bursts temporally overlapped with certain literary and biographical characteristics consistent with mood elevation. Therefore, the combination of genius and bipolar disorder is more likely to explain Li Bai’s literary achievements, rather than the combination of genius and alcohol. Li Bai’s case should no longer be used as a positive example of drinking in the name of “creativity”.

## 1 Introduction

Karl Jaspers posited in General Psychopathology that highly developed psychological realities are uniquely positioned to illuminate universal clinical phenomena. Consequently, individuals with extraordinary cultural and psychological endowments may offer useful material for psychiatric inquiry. Drawing on this paradigm, the present study examines the life and work of the Tang dynasty poet Li Bai (701–762 CE) using retrospective pathography. Rather than presupposing a psychopathological origin of creativity, we evaluate whether data-derived fluctuations in literary productivity temporally coincide with evidence suggestive of mood-spectrum psychopathology (Jaspers, 1963).

Revered as the “Poet Immortal,” Li Bai stands as one of the most prolific and inventive literary figures of the Tang dynasty. Beyond his distinctive artistic style, the pervasive imagery of alcohol in his oeuvre and the documented association between his drinking and his creative output earned him the moniker “Wine Immortal.” Historically, scholars have drawn a strong correlation between his extraordinary creativity and his heavy alcohol consumption, frequently romanticizing his intoxication as an expression of unrestrained bohemian liberty. However, such romanticized accounts fail to acknowledge the underlying psychopathological etiologies and inadvertently minimize the profound clinical risks associated with chronic psychoactive substance use.

A robust body of literature demonstrates an association between literary creativity and selected psychopathological profiles. Notably, in a comprehensive analysis of 1,005 eminent figures, Ludwig found that poets exhibited high rates of substance use, depression, suicide attempts, psychotic experiences, and early-onset mania compared with several other professions (Ludwig, 1992). Subsequent biographical analyses of British and French poets (Martindale, 1972) and 18th-century British writers (Jamison, 1989) corroborate these findings, frequently highlighting bipolar disorder as the most prevalent psychiatric diagnosis within this cohort. Furthermore, these clinical characteristics have been validated in cross-cultural samples. Evaluations of Eastern European authors (Kaufman, 2005) and Hungarian poets confirm that poets consistently demonstrate the highest prevalence of psychiatric morbidity among literary professionals. Notably, up to 67.5% of the Hungarian cohort met the diagnostic criteria for a bipolar spectrum disorder (Czeizel, 2001). Individual pathographical studies have also described bipolar-spectrum symptoms in writers including Johann Wolfgang von Goethe, Sylvia Plath, Virginia Woolf, and Edgar Allan Poe. Li Bai’s surviving oeuvre contains marked shifts between expansive confidence and profound sorrow (Holm-Hadulla et al., 2021; Németh, 2019; Boeira et al., 2017; Gómez, 2012). Accordingly, we tested the hypothesis that quantitatively identified peaks in annual productivity would temporally coincide with literary and biographical evidence of mood elevation, while also examining depressive evidence, alternative situational explanations, and nonpeak years as within-person comparators.

## 2 Methods

### 2.1 Data sources and biographical reconstruction

The primary chronological and biographical sources were *Li Bai’s Complete Collection: Chronological Notes* and Li Changzhi’s *Biography of Li Bai*. Both are Chinese-language sources and were used for dating poems, reconstructing life events, and identifying contemporaneous accounts (An et al., 2015; Li, 2010). English quotations of Li Bai’s and Du Fu’s poetry were taken from published Chinese-English translations by Xu Y and Zhao Y rather than translated by the study authors (Xu, 2021; Zhao, 2020). Using these sources, we reconstructed a chronological timeline and prioritized texts that explicitly described affective states, behavior, or functional consequences.

### 2.2 Quantitative analysis

Adapted from the quantitative pathographical approach used by Gómez in the study of Edgar Allan Poe, we quantified Li Bai’s annual creative output as the number of securely dated poems composed per year according to Li Bai’s Complete Collection: Chronological Notes. We calculated the career mean and SD of annual poem counts and standardized each year as a z score; years exceeding 2 SDs above the mean were designated data-derived high-output peaks (Gómez, 2012). Because the hypothesis concerned episodic within-person change, we also calculated (1) the proportion of all securely dated poems composed during peak years, (2) the mean annual output in peak versus nonpeak years, and (3) the peak-to-nonpeak productivity ratio. These count-based measures are independent of translation and poetic line length. We did not analyze sentence or word length because the fixed metrical conventions of Tang poetry and the semantic properties of classical Chinese make those variables poorly comparable with the English-language metrics used by Gómez. A Spearman rank correlation coefficient was retained to assess the association between age and annual output.

### 2.3 Qualitative analysis

The qualitative phase followed the quantitative analysis in a sequential explanatory design. The three data-derived peaks were used as chronological anchors. Because several symptom-suggestive poems in the source chronology were dated to the year immediately preceding an output maximum, we examined three 2-year windows: 742–743, 752–753, and 758–759 CE. Literary and biographical evidence within each window was mapped to DSM-5 mood-episode symptom domains, including elevated or expansive mood, grandiosity, increased associative or verbal activity, risky behavior, depressed mood, and functional change. The DSM framework was used as a descriptive heuristic rather than as a basis for definitive retrospective diagnosis. Evidence outside these windows was retained only as contextual information when its timing could not be securely linked to a peak. To reduce confirmation bias, we explicitly considered situational explanations for affective changes (e.g., imperial summons, dismissal, exile, pardon, and political upheaval), alcohol-related effects, and evidence inconsistent with a simple peak-equals-hypomania model. We did not classify nonpeak years as “euthymic,” because absence of surviving symptom evidence cannot establish a euthymic baseline in a historical subject.

## 3 Results

### 3.1 Quantitative analysis of creative output

Li Bai lived from 701 to 762 CE, and more than 900 poems survive. Of these, 836 poems had definitive compositional years in Li Bai’s Complete Collection: Chronological Notes and were included in the quantitative analysis. Across the 48-year active career, mean (SD) annual output was 17.42 (16.87) poems (range, 0–73). Three years exceeded 2 SDs above the mean: 743 CE (*n* = 73, *z* = 3.29), 753 CE (*n* = 56, *z* = 2.29), and 759 CE (*n* = 63, *z* = 2.70; Figure 1). Together, these 3 years represented 6.25% of career years but contained 192 of 836 securely dated poems (23.0%). Mean output across peak years was 64.0 poems/year compared with 14.3 poems/year across the other 45 years, yielding a peak-to-nonpeak productivity ratio of 4.47. Annual poem count was positively correlated with age (Spearman *r* = 0.719, *p* < 0.001). Thus, the time series showed both a long-term age-related increase and superimposed episodic bursts; the subsequent qualitative analysis focuses on the three quantitatively identified peaks rather than on symptom-suggestive examples selected independently of the output data.

**Figure 1:**
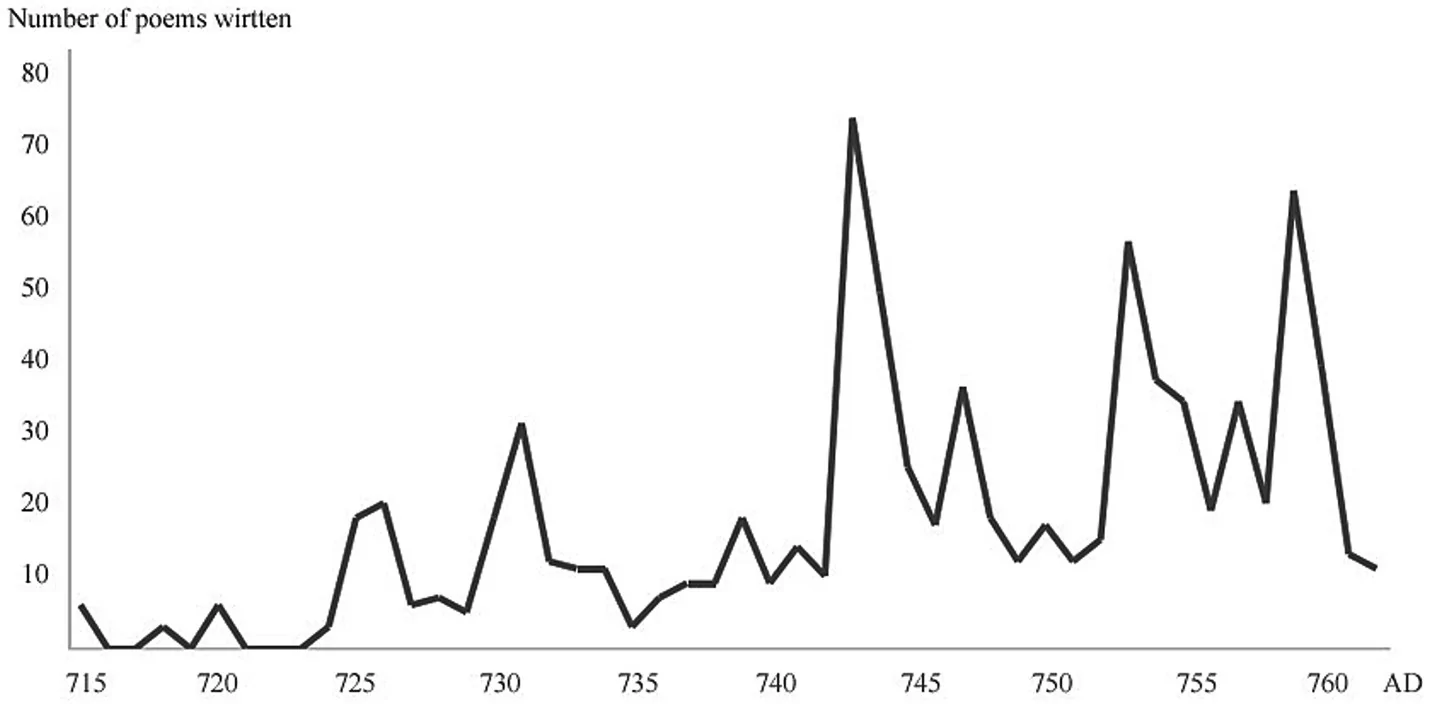
Annual number of securely dated poems written by Li Bai. The three data-derived high-output peaks occurred in 743, 753, and 759 CE (>2 SDs above the career mean).

### 3.2 Episode-centered qualitative analysis

The three peaks shown in Figure 1 were treated as the organizing axis for the qualitative analysis. Table 1 summarizes the dated evidence and the degree to which each item supports, complicates, or weakens a mood-spectrum interpretation.

**Table 1:** Episode-anchored qualitative evidence mapped to DSM-5 mood-symptom domains.

DSM-5-aligned domain	Dated literary/biographical evidence	Peak window	Interpretive constraint
Elevated or expansive mood	742: Departing from My Children at Nanling for the Capital; exuberant departure after imperial summons	742–743	Strong temporal fit, but imperial summons is a plausible situational cause of elation
Grandiose self-appraisal	Court-dismissal period: Hard Is the Way of the World; confidence in exceptional future achievement	742–743	Poetic self-fashioning and conventional rhetoric limit clinical specificity
Expansive sociality / risky behavior	752: Invitation to Wine; celebratory drinking and imagery of selling valuables for wine	752–753	Alcohol intoxication and literary convention are major competing explanations
Depressed or dysphoric mood	753: Farewell to Uncle Yun; sorrow, frustration, and wish to withdraw from worldly affairs	752–753	Occurs within a high-output window, arguing against a one-to-one peak = hypomania model
Elevated affect after major life event	759: Departing from Baidi City at Dawn after imperial pardon; relief and exhilaration	758–759	Pardon from exile provides a strong proportionate nonpathological explanation
Associative / verbal fluency	Puns, phonetic associations, and rapid semantic transitions across the corpus	Not securely episode-linked	Common in classical Chinese poetry; not used to define any peak episode

Episode 1: Imperial Summons, Court Period, and the 743 CE Peak (742–743 CE).

In 742 CE, Li Bai received an imperial summons to Chang’an. Departing from My Children at Nanling for the Capital, dated to that transition, depicts unusually expansive affect and self-appraisal: “Looking up at the sky, I laugh aloud and go. Am I a man to crawl amid the brambles low?” The following year was his largest productivity peak (73 poems; *z* = 3.29). Works associated with the court-dismissal period, including Hard Is the Way of the World, combine frustration with renewed certainty that he would ultimately achieve exceptional political success. Contemporary and retrospective accounts also describe conspicuously uninhibited court behavior. Taken together, the temporal cluster is compatible with elevated mood and grandiosity, but the imperial summons and subsequent court conflict are substantial situational triggers and prevent a purely psychopathological interpretation.

Episode 2: Political Frustration and the 753 CE Peak (752–753 CE).

Invitation to Wine is dated in the study chronology to 752 CE, immediately before the 753 CE output peak (56 poems; *z* = 2.29). Its speaker combines exuberant social drinking, expansive self-confidence (“Heaven has made us talents, we’re not made in vain”), and extravagant spending imagery. In 753 CE, however, Farewell to Uncle Yun also expresses marked sorrow and frustration while retaining passages of soaring ambition. The coexistence of expansive and depressive material within the same high-output window argues against a simple equation of high productivity with a uniformly hypomanic state and is more consistent with affective lability or mixed literary expression. Alcohol intoxication remains a particularly important competing explanation for the behavioral material in this episode.

Episode 3: Exile, Pardon, and the 759 CE Peak (758–759 CE).

The third peak occurred after a qualitatively different sequence of events. Li Bai was traveling toward exile in Yelang in 758–759 CE when he received an imperial pardon in 759 CE. His poem Departing from Baidi City at Dawn, conventionally linked to the return journey after the pardon, conveys rapid movement, relief, and exhilaration. Annual output reached 63 poems (*z* = 2.70). This episode therefore provides temporal convergence between high productivity and elevated affect, but its psychopathological specificity is weaker than in the first two episodes: an unexpected pardon from exile is itself a strong and proportionate reason for elation. We therefore treat the 759 peak as compatible with, but not independently diagnostic of, mood elevation.

### 3.3 Cross-episode symptom mapping and functional context

Across the corpus, evidence potentially relevant to DSM-5 mood-episode domains included elevated or expansive mood, grandiose self-appraisal, associative fluency, and risky or impulsive behavior. However, not all examples could be securely dated to one of the three peak windows. For example, puns, clang associations, and rapid semantic transitions are prominent in Li Bai’s poetry but are also conventional resources of classical Chinese poetics; where precise episode linkage was unavailable, we did not use these features to define a mood episode. Historical descriptions of heavy drinking, extravagant spending, interpersonal conflict at court, and later financial hardship provide functional context but are likewise nonspecific and may reflect personality, social convention, alcohol use, or retrospective embellishment.

The archival evidence does not establish the duration, pervasiveness, or exclusion criteria required for a DSM-defined hypomanic or manic episode. In particular, marked impairment, substance-induced symptoms, and a reliable baseline change cannot be reconstructed with confidence. The symptom mapping in Table 1 is therefore phenomenological and hypothesis-generating rather than diagnostic.

### 3.4 Depressive evidence and the within-person comparator

Li Bai’s corpus also contains explicit depressive and dysphoric material. Farewell to Uncle Yun, dated to 753 CE, states that attempts to drown sorrow in wine only intensify it and expresses a wish to withdraw from worldly affairs. Hard Is the Way of the World contains diminished appetite and frustration alongside later self-confident resolve. These examples are important counterevidence to a binary model in which high-output years are assumed to be hypomanic and low-output years depressive. Accordingly, we did not infer depression from low poem counts and did not label the remaining 45 years “euthymic.” The ipsative comparison is restricted to observable productivity: peak years averaged 64.0 poems/year versus 14.3 poems/year in nonpeak years. Mood-state classification requires independent historical or textual evidence and remains uncertain when such evidence is absent.

## 4 Discussion

Previous epidemiological studies have identified associations between creative occupations and bipolar-spectrum disorders, although the magnitude and direction of these relationships vary across study designs (Kyaga et al., 2013; Kyaga et al., 2011). Literary work may also involve occupational stress, irregular sleep–wake schedules, substance exposure, unstable reinforcement, and financial strain, each of which can interact with mood symptoms (Murray and Johnson, 2010). Furthermore, because poetry typically lacks the structured narrative function of other literary genres, poets derive significantly less emotional catharsis from their writing compared with prose authors (Kaufman and Sexton, 2006). Additionally, most poets experience chronic financial instability. This substantial socioeconomic stress acts as a critical precipitating factor, further increasing the vulnerability of this demographic to psychiatric conditions (Richardson et al., 2016; Richardson et al., 2018). As a professional poet, Li Bai was continuously exposed to this multitude of risk factors, rendering him highly susceptible to bipolar disorder. The present study uses Li Bai as a historical single-case test of temporal convergence rather than as proof that psychopathology causes artistic achievement.

The episode-centered analysis yielded a graded rather than uniform pattern. The 742–743 and 752–753 windows showed the clearest convergence between extreme productivity and literary or biographical features compatible with elevated affect, grandiosity, or reckless behavior. The 752–753 window simultaneously contained prominent dysphoric material, emphasizing that high output cannot be equated with a single mood state. The 758–759 peak coincided with exhilaration after an imperial pardon, but this affective shift has an obvious situational explanation and therefore carries less psychopathological specificity. The quantitative ipsative comparison nonetheless demonstrates a marked concentration of creative production: 23.0% of the securely dated corpus was produced in only 6.25% of career years, and peak-year productivity was 4.47 times the nonpeak-year mean. These findings support the narrower hypothesis that exceptional productivity clustered in discrete periods that sometimes coincided with mood-symptom evidence; they do not establish that bipolar disorder was the cause of those bursts.

Alcohol use is central to the differential interpretation. Bipolar disorder and alcohol use disorder frequently co-occur, and intoxication can alter inhibition, affect, and behavior (Grunze et al., 2021). Experimental evidence suggests that alcohol may reduce executive control without reliably enhancing all components of divergent thinking, whereas bipolar-spectrum traits and states have been associated with selected measures of creative cognition (Johnson et al., 2012; Lloyd-Evans et al., 2006). Divergent thinking involves the capacity to generate multiple unique solutions to open-ended problems. This specific cognitive function significantly influences overall creative performance (Cropley, 2015) Alcohol primarily alters creativity through the suppression of executive control rather than the active facilitation of divergent thinking (Benedek et al., 2017). Conversely, bipolar disorder significantly enhances these divergent cognitive processes (Forthmann et al., 2023). Li Bai’s literary style demonstrates rapid associative fluency and profound semantic divergence. This specific cognitive profile aligns directly with the established pathophysiology of bipolar disorder. Furthermore, alcohol-induced euphoria is characteristically transient and strictly confined to periods of acute intoxication (Brady and Sonne, 1995). In contrast, Li Bai’s creative output exhibits distinct cyclical fluctuations spanning multiple years. This longitudinal episodic chronicity matches the natural disease progression of bipolar disorder. The immediate pharmacokinetic profile of alcohol metabolism cannot explain these prolonged temporal patterns. Finally, chronic alcohol abuse significantly impairs higher cortical functions and induces progressive cognitive decline. This expected deterioration explicitly contradicts Li Bai’s historical record. His annual creative output demonstrated a remarkably consistent upward trend across an active career spanning nearly 50 years. In Li Bai’s case, the multi-year recurrence of productivity peaks is difficult to reduce to acute intoxication alone. Taken together with the literary and biographical characteristics consistent with mood elevation, the combination of his exceptional creative ability and bipolar disorder is more likely to explain his literary achievements than the combination of exceptional creative ability and alcohol. Li Bai’s case should therefore not be used to romanticize drinking as a means of enhancing creativity.

Bipolar disorder may enhance creativity through multiple distinct mechanisms. Guilford identified four cognitive factors significantly correlated with creative thinking. These factors include word fluency, associative fluency, expressional fluency, and ideational fluency (Guilford, 1950). Symptoms of hypomanic or manic episodes directly facilitate these specific cognitive domains. The flight of ideas commonly observed during these states serves as a primary catalyst. Li Bai’s extensive use of puns, clang associations, and rapid semantic associations during his hypomanic periods exemplifies this phenomenon. Furthermore, the increased physical energy and heightened volitional drive characteristic of mood elevations facilitated his prolific literary output (Hankir and Zaman, 2015; Chen and Xiao, 2020; Richards, 1994). The clinical alternation between hypomanic and depressive phases likely enhanced the cognitive integration of positive and negative memory material. This continuous affective cycling provided an extraordinarily rich reservoir of thematic resources for his poetry (Richards, 1994; Isen et al., 1987). Emerging research indicates that bipolar disorder and creativity share fundamental biological processes. Family studies demonstrate that first-degree relatives of individuals with bipolar disorder exhibit significantly higher creativity levels compared with the general population (Kyaga et al., 2011). Genomic analyses further reveal that polygenic risk scores for bipolar disorder and schizophrenia significantly predict individual creativity (Power et al., 2015). Carson’s shared vulnerability model provides an integrative theoretical framework for these empirical observations. This model posits that creative cognition relies on specific operational patterns within dopaminergic and serotonergic neurotransmitter systems. These unique neurobiological profiles enhance creativity by reducing latent inhibition, increasing novelty-seeking behavior, and promoting neural hyperconnectivity (Carson, 2011). Conversely, without sufficient cognitive protective factors, these exact neurotransmitter anomalies elevate the overall risk for severe psychiatric conditions.

## 5 Limitations

This study has several limitations that are particularly important for an ipsative historical design. First, a reliable euthymic baseline cannot be established because no direct mental status examinations or systematic longitudinal symptom ratings exist; consequently, we deliberately refer to “nonpeak” rather than “euthymic” years. Second, surviving poems and biographical records are vulnerable to preservation, dating, and selection biases, and annual poem counts may reflect archival survival as well as true production. Third, the >2-SD threshold is a descriptive rule for identifying unusually productive years, not a formal test of latent mood episodes, and the positive age-output correlation indicates a secular trend superimposed on the bursts. Fourth, qualitative interpretation remains susceptible to confirmation bias. We attempted to mitigate this by anchoring the qualitative review to peaks identified before interpretation, presenting counterevidence within peak periods, and explicitly considering major life events and alcohol as competing explanations. Fifth, many DSM criteria require duration, observable change from baseline, functional consequences, and exclusion of substance-induced states; these elements cannot be verified consistently from the historical record. Finally, some linguistic phenomena discussed in Li Bai’s poetry, including puns and rapid semantic associations, are conventional artistic devices and are not pathognomonic. The findings should therefore be understood as hypothesis-generating evidence of temporal co-occurrence rather than a definitive retrospective diagnosis or causal account of creativity.

## Data Availability

The original contributions presented in the study are included in the article/supplementary material, further inquiries can be directed to the corresponding authors.
